# Supplementary material for: The causal relationship between the human gut microbiota and pyogenic arthritis: a Mendelian randomization study
Source: Front Cell Infect Microbiol. 2024 Nov 26;14:1452480. doi: 10.3389/fcimb.2024.1452480 (PMC11629706; doi:10.3389/fcimb.2024.1452480)
Supplement: Supplementary file 1 [file DataSheet1.zip › mendelian test/ebi-a-GCST90027624.csv_leave_one_out.pdf]

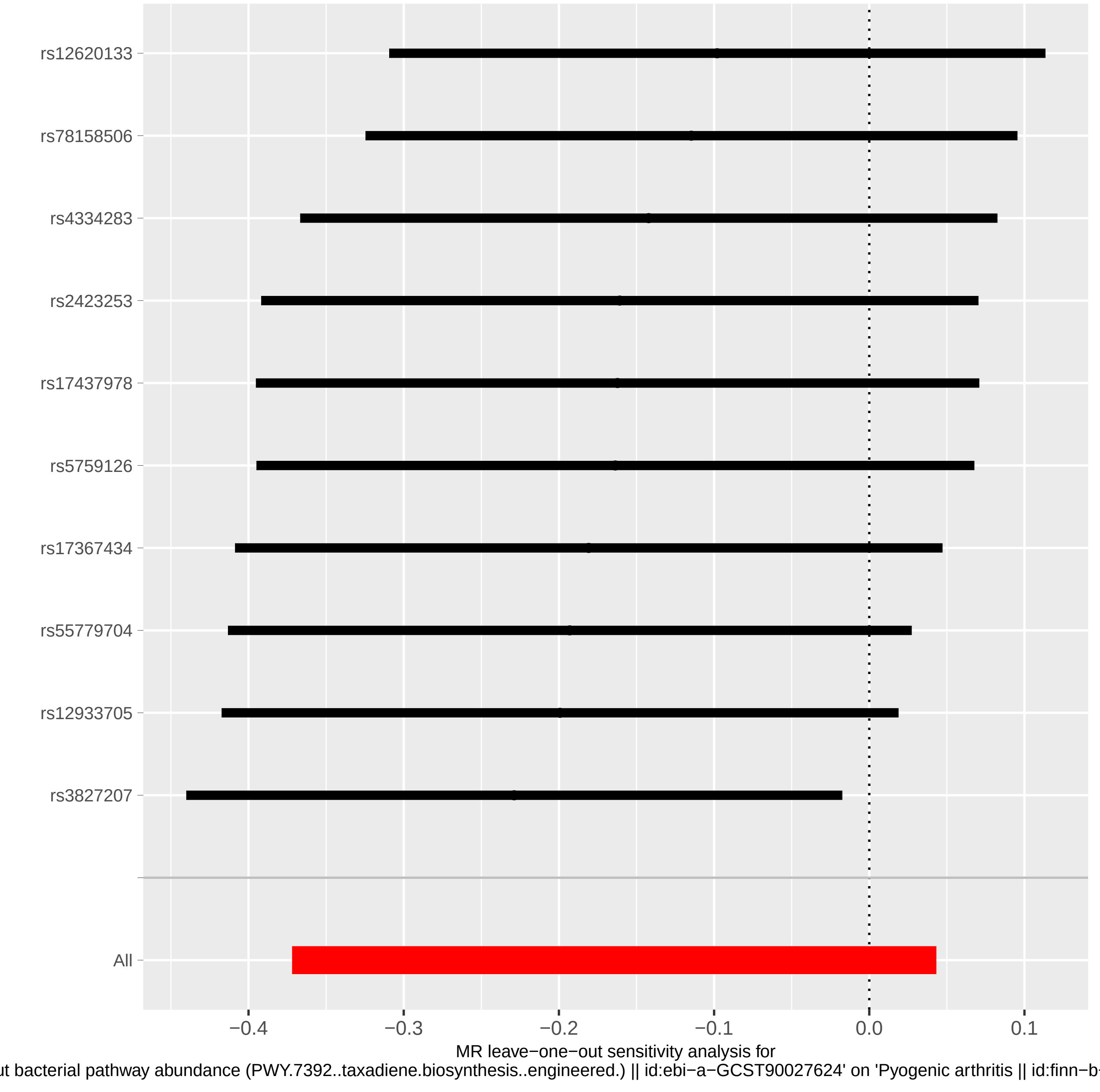

rs12620133
rs78158506
rs4334283
rs2423253
rs17437978
rs5759126
rs17367434
rs55779704
rs12933705
rs3827207
All
−0.4
−0.3
−0.2
−0.1
0.0
0.1
MR leave−one−out sensitivity analysis for
ut bacterial pathway abundance (PWY.7392..taxadiene.biosynthesis..engineered.) || id:ebi−a−GCST90027624' on 'Pyogenic arthritis || id:finn−b
